# Validation of a Questionnaire for Patient Awareness and the Need for a Community-Based Outpatient Antimicrobial Stewardship Program (O-ASP): A Pilot Study

**DOI:** 10.3390/antibiotics10040441

**Published:** 2021-04-15

**Authors:** Sohyun Park, Min Jeong Geum, Hee Jung Choi, Chung-Jong Kim, Won Gun Kwack, Eun Kyoung Chung, Sandy Jeong Rhie

**Affiliations:** 1 Division of Life and Pharmaceutical Sciences Graduate School, Ewha Womans University, Seoul 03760, Korea; sohyun.park@nmc.or.kr (S.P.); MJGEUM@yuhs.ac.kr (M.J.G.); 2 Department of Pharmacy, National Medical Center, Seoul 04564, Korea; 3 Department of Pharmacy, Severance Hospital, Yonsei University Health System, Seoul 03722, Korea; 4 College of Medicine, Ewha Womans University, Seoul 07804, Korea; heechoi@ewha.ac.kr (H.J.C.); erinus@ewha.ac.kr (C.-J.K.); 5 Department of Internal Medicine, Ewha Womans University Mokdong Hospital, Seoul 07985, Korea; 6 Department of Internal Medicine, Ewha Womans University Seoul Hospital, Seoul 07804, Korea; 7 Department of Pulmonary, Allergy and Critical Care Medicine, Kyung Hee University Hospital, Seoul 02447, Korea; wongunnim@naver.com; 8 Department of Pharmacy, College of Pharmacy, Kyung Hee University, Seoul 02447, Korea; 9 Department of Pharmacy, Kyung Hee University Hospital at Gangdong, Seoul 05278, Korea; 10 Department of Pharmacy, Ewha Womans University Mokdong Hospital, Seoul 07985, Korea; 11 College of Pharmacy, Ewha Womans University, Seoul 03760, Korea; 12 Graduate School of Pharmaceutical Sciences, Ewha Womans University, Seoul 03760, Korea

**Keywords:** antimicrobial stewardship program, pharmacist, interprofessional team, outpatient, community pharmacy

## Abstract

An outpatient antimicrobial stewardship program (O-ASP) was developed and implemented to promote appropriate antibiotic therapy in outpatient settings. As active patient involvement is a critical component of an effective O-ASP, this study aimed to develop and validate a questionnaire addressing patient awareness for appropriate antibiotic therapy and the need for pharmaceutical care services (PCS) in the O-ASP in Korea. The questionnaire was drafted based on ASPs and PCS guidelines and validated for content and construct validity using the item-content validity index (I-CVI) and Cronbach’s alpha, respectively. The estimated I-CVI and Cronbach’s alpha were considered excellent or adequate (≥0.8 and 0.70–0.90, respectively) for most of the survey items (17 out of 23 items). The validated questionnaire was utilized in a pilot survey study, including 112 individuals (37% male) with the mean ± SD age of 37 ± 13 years. Among the survey participants, 68% responded that antibiotics had been prescribed appropriately; however, ≥50% showed a lack of knowledge regarding their antibiotic therapy. The participants expressed the need for PCS as part of an O-ASP in the questionnaire (average Likert score ≥3.4/5). In conclusion, our newly validated questionnaire successfully measured patient awareness and knowledge of antimicrobial use and the need for PCS in the O-ASP.

## 1. Introduction

Antibiotic resistance is recognized as one of the biggest threats to global public health [1]. In the United States (US), at least 2.8 million patients are infected annually by an antibiotic-resistant microbial organism, resulting in more than 35,000 infection-related deaths [2]. In South Korea, antimicrobial resistance is a growing problem, with more than 50% of clinical Enterobacteriaceae isolates resistant to at least one antibiotic class [3]. The major risk factor for antibiotic resistance is the inappropriate use of antibiotics [4]. According to previous US studies, more than 50% of antibiotic prescription fills were inappropriate, including at least 30% of unnecessary outpatient antibiotic prescriptions [2,5,6]. A similar prevalence of inappropriate antibiotic use was reported in Korea (30–50%) [7,8]. Antibiotic misuse and overuse are of particular concern in outpatient practice [4] because antibiotic prescriptions are much more prevalent in outpatient settings than in inpatient settings [4,9,10,11]. A recent study published in 2019 reported that >70% of inappropriate antibiotic prescriptions were from outpatient office-based settings, highlighting the prevalent misuse of antibiotics in ambulatory care. Therefore, to minimize the spread of antibiotic resistance accelerated by antimicrobial misuse and overuse, a systematic, organized program for antibiotic use surveillance is crucial in all healthcare settings, including the outpatient practice environment.

An antimicrobial stewardship program (ASP) is developed and implemented to improve and measure appropriate antibiotic use by promoting the selection of the optimal antimicrobial drug regimen concerning dosage, treatment duration, and route of administration [4]. In 2007, the guidelines for developing an institutional ASP were developed with joint efforts of the Infectious Diseases Society of America (IDSA) and the Society for Healthcare Epidemiology of America (SHEA) by addressing the structure of an ASP—inclusion of team members with infectious diseases expertise, physician and pharmacist leadership—and the role of measurement and feedback as critical ASP components [12]. This guideline was most recently updated in 2016 to suggest implementing an ASP across various practice settings, such as emergency department, acute inpatient, and long-term care settings [13]. However, these guidelines have limited generalizability due to the lack of specific recommendations for developing and implementing an ASP in outpatient settings where inappropriate antibiotic use is a serious problem. Later in 2016, the Centers for Disease Control and Prevention (CDC) published recommended core elements of outpatient antibiotic stewardship to address this gap in knowledge and practice [4]. According to these CDC recommendations, relevant clinical partners are critical for a successful outpatient-ASP (O-ASP) in broader healthcare community settings, where community pharmacies and pharmacists are considered as a growing, important venue of healthcare delivery for managing medication therapy as well as educating patients [4].

To develop and implement an effective O-ASP, the CDC recommended active patient involvement to promote appropriate antibiotic use. In fact, patient expectations for antibiotics and possible patient dissatisfaction with clinic visits due to the lack of antibiotic prescription have been recognized as barriers to the improvement of antibiotic prescribing practices, emphasizing targeted educational needs as part of an O-ASP for patients to improve their knowledge and perception regarding appropriate antibiotic use. However, there is a relative paucity of data regarding patient awareness and perception of appropriate antibiotic use, especially in Korea [14,15,16,17]. Moreover, because of its nascency in Korea, the need for and patient acceptance of community pharmacies and pharmacists in the O-ASP have not been evaluated. Considering the variations in healthcare systems and sociocultural aspects across different countries, the feasibility of implementing the recommended core elements of an O-ASP in each country may need to be assessed separately. However, to date, there is no validated questionnaire simultaneously addressing patient awareness and the need for community-based pharmaceutical care services (PCS) of the O-ASP, particularly for Koreans. Therefore, the objectives of this study were (1) to develop and validate a questionnaire addressing patient awareness and the need for community-based PCS of the O-ASP in Korea and (2) to apply our newly developed and validated questionnaire to a pilot survey study.

## 2. Results

### 2.1. Questionnaire Validation

#### 2.1.1. Validation Survey

Overall, 35 (100%) out of 35 respondents returned their completed written survey. Nineteen (54.3%) individuals rated ≥5 out of a 10-point Likert scale for “the items are easy to understand.” However, ten (28.6%) respondents rated ≥5 out of a 10-point Likert scale for “there are too many items in the questionnaire.” In addition, the responses from the survey participants suggested that items 6, 13, 17, 23, 27, and 28 were items that were potentially difficult to answer due to the following reasons: (1) not knowing the name of individual antibiotics, (2) not understanding what preauthorization means, and (3) lack of knowledge regarding pharmaceutical care. Other suggestions from the survey respondents to improve the survey items included “name specific infectious diseases rather than simply stating infection”, “use easy, but specific words”, and “provide examples”.

Based on the validation survey responses, items 6, 13, 17, and 23 were rephrased; items 27 and 28 were deleted. Other items, including 14, were further revised by replacing potentially difficult terms with commonly used words. The items addressing preauthorization were deleted due to their minor role in patient awareness and the need for outpatient PCS to manage infectious diseases.

#### 2.1.2. Content Validity

Overall, the estimated average scale content validity index (S-CVI/Ave) was 0.80 (Table 1). Among the 23 items on patient awareness and the need for community-based PCS in the O-ASP, 17 items (74.0%) showed “excellent” content validity with an item-content validity index (I-CVI) ≥ of 0.8 and κ* > 0.74. In addition, four items (17.4%) were found to have “good” content validity with an I-CVI < 0.8 and κ* ranging from 0.60 to 0.74. However, two items (8.7%) had very low κ* < 0.40 with an I-CVI of 0.5 for both items, suggesting poor content validity.

#### 2.1.3. Construct Validity

Based on the responses from 112 survey participants, the estimated Cronbach’s alpha value of the overall questionnaire for both part 2 and part 3 was 0.797; the calculated Cronbach’s alpha value of part 2 items regarding patient awareness and knowledge of antimicrobial use was 0.702; and for part 3 addressing patients’ need for PCS in an outpatient ASP, the Cronbach’s alpha was estimated to be 0.834. Overall, the calculated Cronbach’s alpha values were in the acceptable range, suggesting adequate internal consistency of our questionnaire.

#### 2.1.4. Scale Dimensionality

The Kaiser–Meyer–Olkin (KMO) measure was 0.82 with *p* < 0.001 from Bartlett’s test of sphericity, suggesting the appropriateness of conducting factor analysis. Factor loading of all items was greater than 0.54. Extraction of principal components using the normalized varimax rotation of variables revealed three factors with an initial eigenvalue greater than 1 (eigenvalues for factors I, II, and III: 5.782, 1.380, and 1.169, respectively), suggesting three dimensions of the scale terms. Part 3 items addressing patients’ need for PCS in an O-ASP were categorized by factors I and II; part 2 items regarding patient awareness of antimicrobial use were represented by factor III (Table 2).

### 2.2. Pilot Survey Analysis

The final, validated questionnaire with 23 items assessing patient awareness and knowledge of antimicrobial use as well as patients’ need for PCS in the O-ASP and 3 demographic items were used in a pilot study. Overall, 112 individuals (37% male) aged 26 to 45 years participated in a pilot survey study and completed the questionnaire. The majority of the survey participants were college graduates (*n* = 69; 63%), followed by high school graduates (*n* = 31; 28%), above-college graduates (*n* = 7; 6%), and below-middle school (*n* = 3; 3%); no responses were obtained from two individuals.

#### 2.2.1. Awareness and Knowledge of Antimicrobial Use

The mean ± standard deviation (SD) Likert scores of participant responses for antibiotic abuse and resistance-related problems were 3.4 ± 1.1 and 3.3 ± 1.1, respectively. Most of the survey participants believed appropriate verification of antibiotic prescriptions had been performed by their pharmacists (mean ± SD Likert score: 3.7 ± 1.1).

Regarding the knowledge of antimicrobial use (Table 3), the majority (68%) of the survey participants believed that antibiotics were prescribed properly (Table 3). In addition, most of the participants knew the reason they were prescribed antibiotics (78%) and when to take their antibiotics (77%). However, only half of the survey participants knew the duration of anti-infective therapy (55%) and the major adverse reactions or precautions (46%). Moreover, the patients knew their antimicrobials by shape and/or color (54%) rather than by antibiotic name (46%). Fewer participants were aware of their antibiotic dosage (38%), the management of antibiotic-induced adverse reactions (25%), and the time for the next follow-up visit after discharge (19%) (Table 3).

#### 2.2.2. Need for Pharmaceutical Care Services in the O-ASP

Overall, the mean Likert scale score for all items was ≥3.4, with the exception of one negatively worded statement, “Pharmacists’ knowledge of antibiotics is irrelevant to the effectiveness of infectious disease treatment” (mean ± SD Likert score: 2.1 ± 0.9) (Table 4). The highest priority was given to the need for pharmacists to validate antimicrobial prescriptions and to monitor resistance status (4.2 ± 0.8). In addition, the survey participants considered the following services of pharmacists to be crucial: providing antibiotic therapy education to other healthcare professionals (4.1 ± 0.8); designing anti-infective therapy based on local infection prevalence and resistance (4.1 ± 0.8), and assisting physicians in choosing the appropriate antibiotic therapy (4.1 ± 0.9). Furthermore, the study subjects expected the active involvement of pharmacists in infectious disease management to reduce the abuse of antimicrobials (3.9 ± 1.0) and to decrease antibiotic resistance (3.8 ± 1.0).

## 3. Discussion

With growing concerns about antibiotic resistance, implementation of an effective ASP is critical to maximizing clinical cure or prevent infection and limiting unintended consequences, such as antibiotic resistance, adverse events, and excessive cost. However, O-ASPs are still not widely implemented due to the relative paucity of evidence-based recommendations compared to institutional ASPs, particularly in Korea. As stated by the CDC guidelines for O-ASPs, patients, as well as community pharmacies and pharmacists, are important parties for a successful O-ASP [4]. Therefore, in this study, we developed and validated a questionnaire assessing patient awareness of antimicrobial use and the need for community-based PCS in the O-ASP based on ASP guidelines as well as PCS guidelines [13,18]. Although many previous studies evaluated the knowledge, attitude, and/or perception regarding antimicrobial stewardship among healthcare professional students and clinicians, such as physicians, nurses, dentists, and pharmacists [19], those assessing patient awareness and the need for pharmacist involvement in the O-ASP were scarce. To our knowledge, our study is the first study to develop and demonstrate the validity of a new questionnaire to investigate patients’ understanding of antimicrobial use and the need for pharmacist involvement in the ASP, particularly in the outpatient setting. According to our pilot survey, the survey participants showed moderate trust in the antibiotics prescribed by their physicians (Table 3). However, in general, patient knowledge of their antibiotic therapy needed to be substantially improved (Table 3). These may account for the need for pharmacist involvement in validating antimicrobial prescriptions, monitoring the emergence of antibiotic resistance, and educating patients and healthcare professionals regarding antibiotic therapy (Table 4). Overall, our pilot survey results suggest that patients consider community-based PCS as a critical component of an O-ASP to improve antimicrobial prescribing appropriateness and their knowledge of antibiotic therapy.

The majority of study participants in our pilot survey (68%) believed that appropriate antibiotics had been prescribed for their current or previous infectious diseases (Table 3). However, a substantial proportion of the patients (32%) considered their antibiotic prescriptions to be inappropriate, which is fairly high compared to a previously reported rate of inappropriate antibiotic prescriptions based on the prevalence of antimicrobial prescription for upper respiratory infection (30% in 2002 to 14% in 2013) [20]. The higher rate of inappropriately prescribed antibiotics perceived by patients might be due to the higher proportion (63%) of female survey participants, who are less likely to trust physicians, as shown in a previous study [21] and the prevalent negative attitudes toward the current healthcare system among Korean patients. In addition, our pilot survey results suggested inadequate knowledge of antimicrobial therapy among the survey participants (Table 3). A substantial portion of the survey participants did not know the antibiotic name, dosage, dosing interval, whether taking it with meals or not and duration of therapy. We postulate that it is because the patients overly trust physicians in general, reflecting our traditional culture and social respect on medical professionals, and too much reliance on the advanced national electronic data network in the healthcare system, which they believe no human mistakes involved in the technology. It could lead them to believe there is no need to know the details about their antibiotic prescription, especially when they face unfamiliar English-origin drug names. This correlates with another finding in our study that survey participants tend to put minimum effort into recognizing their medications by direct visual impression of color and shape instead. Our results were largely consistent with those of previous studies evaluating health literacy among Korean adults as well as medication knowledge in other patient populations [22,23]. According to a previous study investigating health literacy among Korean patients, the average score of health literacy was lower in Korean patients compared to Americans (2.91 out of 6 vs. 3.4 out of 6) [22], despite the higher average literacy and numeracy proficiency of adults in Korea than in the US [24]. Therefore, the lower health literacy, including knowledge of antimicrobial use in Koreans, may not be associated with the general literacy level. Rather, it may be related to barriers to seeking health information, including medication information [22]. Actually, a previous study assessing the association between health literacy and barriers to seeking health information suggested that a lack of patient knowledge of how to get health information could be a significant risk factor for inadequate health literacy [22]. The relatively low health literacy among Korean adults may account for the neutral attitudes of our survey participants regarding antibiotic resistance as well. Therefore, as recommended by the O-ASP guidelines published by the CDC, education for improving health literacy should be provided to patients and their family members as a part of an effective O-ASP to promote appropriate antibiotic use [4].

Because an O-ASP should cover a broader range of the healthcare community, close collaboration is critical among clinicians, clinic leaders, patients, and various healthcare sectors, including acute care hospitals, long-term care facilities, government health departments, health insurance companies, healthcare professional societies, local microbiologic laboratories, and community pharmacies [4]. The CDC guidelines for outpatient antimicrobial stewardship indicated community pharmacies and pharmacists as a trusted source of healthcare information—located near clinics or hospitals where patients are frequently seen—for the management of common infectious diseases [4]. Community pharmacists can contribute to the O-ASP by providing patient recommendations for nonprescription medications for symptomatic relief, facilitating medication therapy management, screening patients for drug interactions and allergies, and educating patients regarding appropriate antibiotic use and anticipated side effects [4]. According to a previous study by Bishop and colleagues, these antibiotic stewardship interventions led by community pharmacists could be performed via the following strategies: collaborative practice agreements (CPA), point-of-care (POC) testing, patient counseling, academic detailing, and advocacy for patients and other healthcare practitioners [25]. However, ASP activities of community pharmacists may vary greatly in different countries due to substantial differences in the legal scope of practice, available resources, healthcare delivery system, and patient expectations [26,27]. Based on our pilot survey responses, Korean patients expected and needed active pharmacist involvement in infectious disease management to improve overall treatment outcomes (Table 4). Specifically, the main expectations of patients in the O-ASP were for the pharmacists to play advocating or consulting roles in antibiotic therapy [25]. Patients expected less contribution of pharmacists in reducing antimicrobial abuse or resistance or in saving antibiotic treatment cost through pharmacist-led interventions because these activities might require CPA or POC testing that are either uncommon or outside the legal scope of pharmacists’ practice in Korea [25]. Overall, our pilot survey using the newly developed and validated questionnaire showed an essential patient need for community-based PCS to improve appropriate antimicrobial use and, ultimately, to optimize treatment outcomes of infectious diseases.

Globally, O-ASP is still at a nascent stage, particularly in Korea, with several barriers to development and implementation. One of the major challenges is a gap between clinician knowledge of best practices and recommended practices by clinical guidelines [4]. This may account for the relatively low rate (<70%) of the perceived appropriateness of antibiotic prescribing among patients, as in our present study (Table 3). According to a recent study conducted in Korea, approximately ≤50% of physicians were aware of the standard duration of antibiotic therapy recommended by the clinical practice guidelines [19]. Similar results have been published in other countries regarding the knowledge of physicians concerning appropriate antimicrobial use, with the antimicrobial knowledge score ranging from 0% to 80% [28,29]. This relatively high prevalence of inappropriate antibiotic prescribing might be associated with clinician perception of patient expectations for antibiotics and/or clinician concerns about decreased patient satisfaction with clinical visits when antibiotics are not prescribed [4]. Indeed, a previous survey study on the Korean general population (*n* = 547) showed that 40.2% to 83.2% of the survey participants expected an antibiotic prescription from the physician in the presence of respiratory symptoms [30]. Similarly, 73% of physicians (*n* = 409) participating in another survey study reported perceived expectations for antibiotics from parents of children with respiratory symptoms [31]. Overall, this highlights the urgent need for patient involvement in educational programs on appropriate antibiotic use, including clinical situations where antibiotics are needed and the potential risks of antimicrobial therapy [4]. As recommended by the CDC, community pharmacies and pharmacists are a trusted source of providing healthcare information and patient education regarding appropriate antibiotic use and anticipated adverse events [4]. The need for patient education regarding appropriate antibiotic use was emphasized in our present study showing a relative lack of patient knowledge of antimicrobial therapy (Table 3). Considering that medication education and counseling are provided to patients by community pharmacists as a core responsibility, our current findings may suggest a need for improvement of existing programs rather than the need for development and implementation of a new patient education program. Actually, in a previous survey study conducted in Korea by Yang and colleagues, only 34% of patients (*n* = 252) were satisfied with medication counseling at community pharmacies, primarily due to insufficient counseling time [32] associated with the perceived lack of time by community pharmacists. Therefore, targeted educational programs and committed personnel are required to develop and implement a successful ASP in outpatient settings. Further studies are necessary to evaluate interventions for improving antibiotic stewardship efforts, such as feedback or incentives provided by payers or government health departments [33].

Our study has some limitations. First, although we confirmed the overall validity and reliability of our newly developed questionnaire, our pilot survey results should be carefully interpreted due to the limited number of survey participants. Future large-scale studies are warranted for robust responses using our validated questionnaire. Additionally, caution needs to be exercised when interpreting our pilot survey results due to the difference in the characteristic demographic distribution between our current study sample and the general Korean population. Specifically, given that the mean age of the general Korean population was 43 years, the majority of our pilot survey participants with a mean age of 37 years were relatively young [34]. Actually, only 5% of our pilot survey participants were over 65 years old, clearly limiting the generalizability of our pilot survey study findings. Because older age is a risk factor for various infectious diseases [35], elderly individuals are more likely to receive antimicrobial therapy. Thus, our results may under-represent the educational needs and the demand of the O-ASP and associated PCS for elderly patients. Thus, further studies are needed to investigate the responses primarily from elderly people who are susceptible to infectious diseases. Moreover, the pilot survey results may have a limit to generalizing findings beyond a Korean population, which should be required careful interpretation. Second, there may be concerns of a small sample size and a potential selection bias in the pilot study. Thus, to avoid those limitations, we had minimum inclusion criteria. Moreover, two independent surveyors were blinded to the specific details of the study and patient case and they were not involved in further processes during the study. Lastly, compared with a 5-point Likert scale, a 7-Likert scale produces better distributions of data without having to maintain too many response options. It tends to provide a more accurate, easier use and a better reflection of a respondent’s true evaluation. When considering our study with the small number of survey respondents and a large number of items (26 items) in our study, we used a 5-point Likert scale, which would benefit from providing an increase of response rate and response quality along with reducing respondents’ selection frustration. However, a 7-Likert scale needs to be considered with a further study with large-scale survey respondents. Overall, despite these limitations that require our findings to be interpreted with caution, the questionnaire developed and tested in this study sample could be a promising tool for designing ASPs to monitor and regulate antimicrobial agent use.

## 4. Methods

### 4.1. Development of the Questionnaire

The draft items were brainstormed and prototyped based on research team discussions; clinician expert opinions; and previous literature, including treatment guidelines published by the IDSA and SHEA [13], the PCS guidelines by the American Society of Health-System Pharmacists (ASHP) [18], and other scientific articles published in peer-reviewed journals [36,37,38,39].

The initially drafted questionnaire consisted of three parts, including a total of 28 items: part 1 for demographic characteristics of survey participants (3 items), part 2 for patient awareness (3 items with a 5-point Likert scale) and knowledge (10 items with yes/no responses) of antimicrobial therapy, and part 3 for the need for PCS of an O-ASP (12 items using a 5-point Likert scale).

### 4.2. Validation of the Questionnaire

#### 4.2.1. Validation Survey

The drafted questionnaire was administered to a convenience sample of 35 lay individuals who agreed to participate in the survey and satisfied the inclusion criteria of current or previous use of antimicrobials for ≥3 days. At the end of the draft questionnaire, the survey participants were asked about (1) how clear the survey items were and (2) how difficult each item was to answer using free text responses as well as 10-point Likert scales. The collected responses of the validation survey were analyzed to identify issues and problems associated with the drafted questionnaire. The survey items with issues and/or problems were revised by replacing words, deleting the irrelevant phrases, and rephrasing sentences to improve the questionnaire. After the revision, further validation was conducted to develop the final questionnaire and perform a pilot survey study.

#### 4.2.2. Content Validity

The revised questionnaire was evaluated for content validity index (CVI) [40] by 10 experts, including four pharmacists, three physicians, a nurse, a clinical research associate, and a faculty member from the Department of Public Health. The experts were asked to rate each item in terms of clarity and relevance to the construct by scoring on a 4-point ordinal scale (1, not relevant; 2, somewhat relevant; 3, quite relevant; 4, highly relevant).

I-CVI for all items was calculated by computing modified kappa (κ*) using the numerical values of probability of chance agreement and CVI of each item (I-CVI) (Equations (1) and (2)). The S-CVI was also calculated as an average of I-CVIs for all items of the scale (S-CVI/Ave). CVIs were considered excellent when the I-CVI was ≥0.8.

(1) $$\mathrm{Item}\;\mathrm{content}\;\mathrm{validity}\;\mathrm{index}\;(\mathrm{I-CVI}):\;\mathrm{I-CVI}\;\frac{\mathrm{the}\;\mathrm{numbers}\;\mathrm{of}\;\mathrm{experts}\;\mathrm{giving}\;a\;\mathrm{rating}\;\mathrm{of}\;3\;\mathrm{or}\;4}{\mathrm{the}\;\mathrm{numbers}\;\mathrm{of}\;\mathrm{experts}}$$

(2) $$\mathrm{kappa}\;\mathrm{designating}\;\mathrm{agreement}\;\mathrm{on}\;\mathrm{relevance}\;(\kappa^{*}):\;\kappa *=\frac{\left(\mathrm{I-CVI}-\mathrm{Pc}\right)}{\left(1-\mathrm{Pc}\right)}$$

where probability of chance occurrence (−P_c_) = [N!/A!(N − A)!] × 0.5 N, N = number of experts, and A = number agreeing on good relevance. Kappa was considered fair if κ* was 0.40–0.59, good if 0.60–0.74, and excellent if >0.74. S-CVI/Ave was the mean value of I-CVI.

Internal consistency was evaluated based on Cronbach’s alpha and factor analysis utilizing the responses collected in the pilot survey study as a scale reliability measure.

#### 4.2.3. Construct Validity

Internal consistency of part 2 and part 3 was assessed using Cronbach’s alpha, which describes the extent to which all the items in a questionnaire measure the same concept or construct. According to the guidelines, Cronbach’s alpha is interpreted as unacceptable if <0.60; undesirable if 0.60 to <0.65; minimally acceptable if 0.65 to <0.70; respectable if 0.70 to <0.80; excellent if 0.80 to <0.90; excessive consistency implying item redundancy if ≥0.90 [40]. In this study, a reliability coefficient of ≥0.70 was considered adequate.

#### 4.2.4. Scale Dimensionality

Factor analysis was performed to investigate the dimensionality of the scale. Principal component factor analysis with subsequent varimax rotation was used to derive factors potentially corresponding to the subscales for the three parts in the questionnaire. Prior to extracting the factors, the KMO measure and Bartlett’s test of sphericity were conducted to examine the appropriateness of factor analysis with the respondent data [41]. The KMO index ranges from 0 to 1, with 0.50 considered appropriate for factor analysis. Bartlett’s test of sphericity should be significant (*p* < 0.05) to appropriately perform factor analysis. Factors were retained if the eigenvalue corresponding to the factor was ≥1.0. Individual items loading onto a factor with a factor loading of ≥0.54 were assigned to that factor.

### 4.3. Pilot Survey Study

A cross-sectional survey was conducted from November 2017 to March 2018 with a convenience sample of outpatients or their caregivers for minor patients in Seoul, Daegu (the southern part of Korea), and Gyeonggi-do (the vicinity of Seoul). Included individuals were those with a previous history of or currently receiving antimicrobial therapy for infectious diseases for ≥3 days. Informed consent was obtained from each individual prior to participation in the study. The study was approved by the Institutional Review Board at Ewha Womans University (No. 143-7).

Descriptive statistics were used to characterize responses of the study participants regarding their awareness of appropriate antimicrobial use and the need for community-based PCS in the O-ASP. All statistical analyses, including internal consistency and factor analysis, were performed with SPSS version 23 (IBM Corporation, Armonk, NY, USA).

## 5. Conclusions

In conclusion, we developed a survey questionnaire measuring patient awareness and knowledge of antimicrobial use and the need for PCS in the O-ASP based on the ASP guidelines and PCS guidelines. The newly developed questionnaire was validated concerning content validity and internal consistency. Utilizing this validated questionnaire in a pilot survey study, we successfully assessed patient awareness and knowledge regarding appropriate antibiotic therapy and the patients’ need for PCS in the O-ASP to improve infectious disease pharmacotherapy. The relative lack of knowledge and awareness of appropriate antimicrobial therapy revealed by our pilot survey indicated a substantial need for PCS to improve outpatient antibiotic therapy. A systematic, organized approach, such as an O-ASP, must be developed and implemented in outpatient settings for optimal infectious disease pharmacotherapy in Korea.

## Figures and Tables

**Table 1:** The items in the questionnaire and content validity based on the responses from ten experts; 5-point Likert scale from strongly disagree (1) to strongly agree (5).

Item #	Statement	I-CVI ^1^	κ* ^2^	Interpretation ^3^
**Part 1. Demographics**				
1	Sex	Not applicable		
2	Age	Not applicable		
3	Education level	Not applicable		
**Part 2a. Awareness of antimicrobial use (5-point Likert scale)**				
4	Antimicrobial agents are nationally abused.	0.8	0.79	Excellent
5	Antibiotic resistance is a nationwide problem.	0.7	0.66	Good
6	Pharmacists properly check the reason to take antimicrobials, their dose and duration of use; monitor their side effects; and provide counseling regarding my antibiotics.	0.8	0.79	Excellent
**Part 2b. Knowledge of antimicrobial use (yes/no items)**				
7	I believe anti-infectives are properly prescribed to me.	0.8	0.79	Excellent
8	I know (knew) the name of my antibiotics.	0.9	0.90	Excellent
9	I know (knew) the reason for taking my antibiotics.	1.0	1.00	Excellent
10	I know (knew) the dose of my antibiotics.	0.5	0.34	Poor
11	I know (knew) the duration of my antimicrobial therapy.	0.9	0.90	Excellent
12	I know (knew) the shape or color of my antibiotics.	0.7	0.66	Good
13	I know (knew) when to take my antibiotics.	0.9	0.90	Excellent
14	I know (knew) the adverse reactions or precautions related to my antibiotics.	1.0	1.00	Excellent
15	I know (knew) how to manage the adverse reaction caused by my anti-infectives.	0.8	0.79	Excellent
16	I know (knew) the time for the next follow-up visit after discharge from the hospital^.4^	0.5	0.34	Poor
**Part 3. Patient need for community-based pharmaceutical care services in the outpatient antimicrobial stewardship program** **(5-point Likert scale)**				
17	The abuse of antimicrobials would be reduced by the active involvement of pharmacists in infectious disease management.	0.8	0.79	Excellent
18	Antibiotic resistance would be decreased by the active involvement of pharmacists in infectious disease management.	0.8	0.79	Excellent
19	Treatment outcomes of infectious diseases would be improved with stronger antimicrobial knowledge of pharmacists.	0.8	0.79	Excellent
20	Pharmacists’ knowledge of antibiotics is irrelevant to the effectiveness of infectious disease treatment.	0.7	0.66	Good
21	Pharmacists’ intervention helps the physician choose the appropriate antimicrobial therapy.	0.8	0.79	Excellent
22	Pharmacists’ intervention prevents excessive antibiotic prescriptions.	0.7	0.66	Good
23	Pharmacists’ intervention saves antimicrobial drug costs.	0.8	0.79	Excellent
24	Pharmacists need to validate antibiotic prescriptions and monitor resistance status continuously.	1.0	1.00	Excellent
25	Treatment outcomes of infectious diseases would be improved by pharmacy education for other healthcare professionals.	0.9	0.90	Excellent
26	Consideration of local infection prevalence and resistance patterns would improve antimicrobial treatment outcomes.	0.8	0.79	Excellent
Overall, content validity		S-CVI/Average ^5^ = 0.8		

^1^ I-CVI (item content validity index) = the number of experts giving a rating of 3 or 4/the number of experts. ^2^ κ* = kappa designating agreement on relevance: κ* = (I-CVI ‒ P_c_)/(1 − P_c_),where ‒ P_c_ (probability of a chance occurrence) = [N!/A!(N − A)!] × 0.5 ^N^, N = number of experts, and A = number agreeing on good relevance. ^3^ Interpretation criteria for κ*: poor = κ* < 0.40; fair = κ* of 0.40‒0.59; good = κ* of 0.60‒0.74; excellent = κ* > 0.74. ^4^ Designed to be answered only by patients, who had been hospitalized previously. ^5^ S-CVI/Ave (average scale content validity index) = mean of I-CVI.

**Table 2:** Factor analysis of the 5-point Likert-scale items in parts 2 and 3 of the questionnaire ^1^.

(A) Results of the varimax rotation						
**PA**	**Initial Eigenvalues**			**Rotation Sums of Squared Loadings**		
	**Total**	**% of Variance**	**Cumulative%**	**Total**	**% of Variance**	**Cumulative%**
I	5.782	36.3	48.182	3.398	28.315	28.315
II	1.380	10.6	59.684	2.779	23.159	51.474
III	1.169	7.6	69.427	2.154	17.952	69.427
(B) Component matrix and internal consistency of each factor **^2^**						
**Questionnaire Part**		**Item** **no.**	**Factor**			**Internal Consistency**
			**I**	**II**	**III**	**Cronbach’s α**
Part 3		Q23	**0.831**	0.099	0.316	0.840
		Q26	**0.787**	0.163	0.318	
		Q24	**0.701**	0.360	0.021	
		Q22	**0.694**	0.197	0.004	
		Q25	**0.632**	0.090	0.163	
Part 3		Q18	0.031	**0.908**	0.063	0.793
		Q17	0.314	**0.855**	0.050	
		Q19	0.327	**0.651**	0.393	
		Q21	0.419	**0.542**	0.498	
Part 2		Q6	0.036	−0.032	**0.879**	0.702
		Q4	0.258	0.249	**0.643**	
		Q5	0.515	0.481	**0.575**	

^1^ One negatively worded item in part 3 (item #20) was excluded from the factor analysis due to redundancy. ^2^ Bolded values indicate item loadings greater than the threshold for assigning an item to a factor, which was set to 0.54.

**Table 3:** Knowledge of antimicrobial use among survey participants (*n* = 112).

Item	Yes (*n* (%))	No (*n* (%))
I believe anti-infectives are properly prescribed to me. ^1^	76 (68)	35 (31)
I know (knew) the name of my antibiotics.	51 (46)	61 (54)
I know (knew) the reason for taking my antibiotics. ^1^	87 (78)	24 (21)
I know (knew) the dose of my antibiotics.	43 (38)	69 (62)
I know (knew) the duration of my antimicrobial therapy.	62 (55)	50 (45)
I know (knew) the shape or color of my antibiotics.	60 (54)	52 (46)
I know (knew) when to take my antibiotics.	86 (77)	26 (23)
I know (knew) adverse reactions or precautions of my antibiotics.	51 (46)	61 (54)
I know (knew) how to manage the adverse reactions caused by my anti-infectives.	28 (25)	84 (75)
I know (knew) the time for the next follow-up visit after discharge from the hospital. ^2^	21 (19)	35 (31)

^1^ One missing response was excluded. ^2^ Only 56 study participants were prescribed antibiotics after hospital discharge.

**Table 4:** Patient need for community-based pharmaceutical care services in the outpatient antimicrobial stewardship program (*n* = 112); 5-point Likert scale from strongly disagree (1) to strongly agree (5).

Statement	Mean ± SD
The abuse of antimicrobials would be reduced by the active involvement of pharmacists in infectious disease management.	3.9 ± 1.0
Antibiotic resistance would be decreased by the active involvement of pharmacists in infectious disease management.	3.8 ± 1.0
Treatment outcomes of infectious diseases would be improved with stronger antimicrobial knowledge of pharmacists.	4.0 ± 1.0
Pharmacists’ knowledge of antibiotics is irrelevant to the effectiveness of infectious disease treatment.	2.1 ± 0.9
Pharmacists’ intervention helps the physician choose appropriate antimicrobial therapy.	4.1 ± 0.9
Pharmacists’ intervention prevents excessive antibiotic prescriptions.	3.7 ± 1.0
Pharmacists’ intervention saves antimicrobial drug costs.	3.4 ± 1.1
Pharmacists need to validate antibiotic prescriptions and monitor resistance status continuously.	4.2 ± 0.8
Treatment outcomes of infectious diseases would be improved by pharmacy education for other healthcare professionals.	4.1 ± 0.8
Consideration of local infection prevalence and resistance pattern would improve antimicrobial treatment outcomes.	4.1 ± 0.8

## Abbreviations

O-ASP	Outpatient antimicrobial stewardship program
ASP	Antimicrobial stewardship program
ASHP	American Society of Health-System Pharmacists
CDC	Centers for Disease Control and Prevention
CPA	Collaborative practice agreements
CVI	Content validity index
I-CVI	Item-content validity index
IDSA	Infectious Diseases Society of America
KMO	Kaiser–Meyer–Olkin
PCS	Pharmaceutical care services
POC	Point-of-care
S-CVI/Ave	Average scale content validity index
SHEA	Society for Healthcare Epidemiology of America
US	United States
